# Endocardial Remodeling in Heart Failure Patients with Impaired and Preserved Left Ventricular Systolic Function-A Magnetic Resonance Image Study

**DOI:** 10.1038/srep20868

**Published:** 2016-02-15

**Authors:** Lian-Yu Lin, Mao-Yuan M. Su, Van-Truong Pham, Thi-Thao Tran, Yung-Hung Wang, Wen-Yih I. Tseng, Men-Tzung Lo, Jiunn-Lee Lin

**Affiliations:** 1 Department of Internal Medicine, National Taiwan University Hospital, Taipei, Taiwan; 2 Department of Medical Imaging, National Taiwan University Hospital, Taipei, Taiwan; 3 Institute of Translational and Interdisciplinary Medicine and Department of Biomedical Sciences and Engineering, National Central University, Chungli, Taiwan; 4 Department of Electrical Engineering, National Central University, Chungli, Taiwan; 5 Center for Optoelectronic Biomedicine, National Taiwan University College of Medicine, Taipei, Taiwan

## Abstract

Left ventricular (LV) trabeculation has been studied in certain forms of cardiomyopathy. However, the changes of LV endocardial trabeculation during the remodeling process leading to heart failure (HF) are unclear. Seventy-four patients with systolic heart failure (SHF), 65 with heart failure with preserved ejection fraction (HFpEF) and 61 without HF were prospectively enrolled. All subjects received magnetic resonance imaging (MRI) study including cine, T1 and late gadolinium enhancement (LGE) images. Trabecular-papillary muscle (TPM) mass, fractal dimension (FD) and extracellular volume (ECV) were derived. The results showed that TPM mass index was higher in patients with SHF than that in patients with HFpEF and non-HF. The TPM mass-LV mass ratio (TPMm/LVM) was higher in SHF group than that in HFpEF and non-HF. FD was not different among groups. The presence of LGE was inversely associated with TPM mass index and TPMm/LVM while the ECV were positively associated with TPMm/LVM. The FD was positively associated with LV chamber size. In conclusion, TPM increases in patients with SHF and are probably related to myocardial cell hypertrophy and fibrotic repair during remodeling. The FD increases with the dilatation of LV chamber but remain unchanged with the deterioration of LV function.

Patients with heart failure (HF) carry an excess risk of morbidity and mortality, making it a major public health concern^1^. Ventricular remodeling, a predecessor of HF, refers to the changes in size, shape, structure and function of the heart after injury to the myocardium and is most commonly seen in patients after large myocardial infarction (MI) and in those with dilated forms of cardiomyopathy^2^. Previously, most of the observations of ventricular remodeling focused on specific forms of gross pathologic changes including increased left ventricular (LV) volume and perturbation in the normal elliptical LV chamber configuration. The changes of LV trabeculation in the process of remodeling have seldom been investigated.

In some forms of cardiomyopathy, hypertrabeculation has been associated with adverse cardiovascular outcomes^3^,^4^. In healthy hearts, trabecular phenotypes are hard to define because of their large variation and interethnic differences^5^,^6^. Recently, some reliable methods have been developed to quantify LV trabeculation. In the Framingham Offspring cohort, trabeculae and papillary muscle (TPM) mass and LV mass (LVM) ratio (TPMm/LVM) derived from cardiac magnetic resonance imaging (MRI) has been found to inversely correlate with age and loading conditions^7^. In a substudy of Multi-Ethnic Study of Atherosclerosis (MESA), fractal dimension (FD), a measure of endocardial complexity is positively associated with hypertension and LVM^8^. Since these studies included subjects with relative normal heart function, these results imply that subtle LV trabeculation remodeling, revealed by novel quantitative technique, actually takes place in early stage of LV dysfunction.

Myocardium remodeling starts with injury. When the pathological processes take effect, the activation of myofibroblasts and the increase in collagen synthesis result in two major types of fibrosis, reactive interstitial fibrosis and replacement fibrosis, commonly affect diastole first and subsequently involve systolic performance^9^. Reactive fibrosis or interstitial fibrosis has mostly been described in hypertension, diabetes mellitus (DM), aging heart and idiopathic dilated cardiomyopathy where the activation of renin-angiotensin aldosterone system, beta-adrenergic system and the excess of reactive oxygen species are major contributors. Replacement fibrosis corresponds to the replacement of myocytes after cell necrosis by plexiform fibrosis and is mostly seen in patients with previous myocardial infarction or myocarditis^10^. Even though late gadolinium enhancement (LGE) image is a sensitive and reproducible method to detect and quantify replacement fibrosis but it’s sensitivity is limited for the assessment of diffuse interstitial fibrosis. Previously, the only methodology available to assess myocardial diffuse interstitial fibrosis was the histopathology assessment of endomyocardial tissue biopsy specimen. Recently, diffuse myocardial interstitial fibrosis has been proved to be able to be quantitatively defined by MRI contrast-enhanced T1 mapping technique and could be reliably quantified by extracellular volume fraction (ECV)^11^. Through this noninvasive technique, diffuse myocardial interstitial fibrosis could be broadly studied in clinical settings.

In this study, we plan to investigate the trabeculation of LV in a group of HF patients including systolic HF (SHF) and HF with preserved ejection fraction (HFpEF) by using non-HF patients as controls. Our goal is to study the changes of LV trabeculation in the remodeling process leading to HF and delineate its mechanisms related to myocardial fibrosis.

## Results

Sequential 74 patients with SHF, 65 patients with HFpEF, and 61 patients without HF were enrolled in the study. The demographics of the study population were summarized in Table 1. The age was younger in SHF group as compared with that in HFpEF group (64.0; IQR: 55.0**~**71.3 vs. 71.0; IQR: 60.0**~**77.5, P = 0.003) and there were more male patients in the SHF group compared to HFpEF (83.8% vs. 46.2%, P < 0.001) and non-HF (83.8% vs. 45.9%, P < 0.001) groups. Patients with SHF had higher body mass index (BMI) than that in non-HF group (24.6; IQR: 22.6**~**26.9 vs. 26.5; IQR: 25.0**~**29.6, P = 0.001). Patients with SHF and HFpEF had higher rates of prior MI than patients without HF (25.7% and 18.5%, vs. 4.9%, P = 0.001 and 0.026 respectively). More patients in SHF group received beta-blocker treatment as compared with that in HFpEF (71.6% vs. 47.7%, P = 0.005) and non-HF (71.6% vs. 49.2%, P = 0.012) groups. The other demographics were not statistically different.

The MRI parameters of the three groups were shown in Table 2. LV volume and mass indices including left ventricular end-diastolic volume index (LVEDVi), left ventricular end-systolic volume index (LVESVi) and left ventricular mass index (LVMi) were all significantly higher in the SHF group as compared to that in HFpEF and non-HF groups. The LV volume and mass indices were not different between HFpEF and non-HF groups. The chance of LGE was higher in patients with SHF and HFpEF than that in non-HF group (67.1% and 41.5% vs. 14.8%, P < 0.001 and P = 0.001, respectively). The chance of LGE was also higher in patients with SHF than that in HFpEF (67.1% vs. 41.5%, P = 0.003). For myocardial diffuse fibrosis, patients with SHF had significantly higher ECV than patients with HFpEF (32.5%; IQR: 28.6**~**43.1% vs. 30.7%; IQR: 28.6**~**32.5%, P = 0.003) and without HF (32.5%; IQR: 28.6**~**43.1% vs. 27.8%; IQR: 25.6**~**29.3%, P < 0.001). The mean ECV was also significantly higher in patients with HFpEF than that in patients without HF (30.7%; IQR: 28.6**~**32.5% vs. 27.8%; IQR: 25.6**~**29.3%, P < 0.001). For TPM, the TPM mass index was higher in patients with SHF than that in patients with HFpEF (24.1 g; IQR: 18.6**~**31.3 g vs. 13.8 g; IQR: 11.9**~**17.7 g, P < 0.001) and without HF (24.1 g; IQR: 18.6**~**31.3 g vs. 13.3 g; IQR: 11.2**~**17.9 g, P < 0.001). After normalization of TPM by LVM, the TPMm/LVM was higher in SHF group than that in HFpEF (25.7; IQR: 22.0**~**29.8 vs. 23.7; IQR: 20.1**~**25.2, P < 0.001) and non-HF (25.7; IQR: 22.0**~**29.8 vs. 23.1; IQR: 20.4**~**25.0, P < 0.001) groups. The trabecular complexity indicator, FD, was not different among three groups.

The correlation coefficients (C.C) as well as their confidence interval (CI) of the multiple linear regression analyses were shown in Table 3. As demonstrated in Table 3, For TPM mass index, among the covariates, male gender (3.313; 95% CI: 0.326**~**6.300, P = 0.030) and the highest tertile of LVMi (9.924; 95% CI: 5.984**~**13.864, P < 0.001) were associated with higher TPM mass index while the presence of LGE (−5.308; 95% CI: −8.482**~**−2.134, P = 0.001) was inversely associated with TPM mass index. For TPMm/LVM, the presence of LGE was inversely associated with TPMm/LVM (−0.026; 95% CI: −0.051**~**−0.002, P = 0.035) while the highest tertile of ECV was positively associated with TPMm/LVM (0.035; 95% CI: 0.006**~**0.063, P = 0.017). For FD, higher LVEDVi (0.028; 95% CI: 0.010**~**0.045, P = 0.002 for T2 vs. T1 and 0.056; 95% CI: 0.026**~**0.086, P < 0.001 for T3 vs. T1) was associated with higher FD.

## Discussion

In this study, we demonstrated that both TPM mass index and TPMm/LVM increase in patients with SHF. From the regression analyses, the TPM mass index increases with greater LVMi while the TPMm/LVM increases with greater ECV or interstitial fibrosis. We also showed that both TPM mass index and TPMm/LVM decrease with the presence of LGE. These findings are compatible with two forms of fibrotic repair during LV remodeling. The myocardium is composed for about the 70% of its volume by parallel cardiac muscle cells, embedding in an extracellular matrix network which forming the remaining 30% of the volume^12^,^13^. In physiological condition, the fibrillary collagen network is in intimate contact with myocardial cells and plays a critical role in the maintenance of ventricular shape and function^10^. When pathological processes take effect, the activation of myofibroblasts and the increase in collagen synthesis result in two major types of fibrosis, replacement fibrosis and reactive interstitial fibrosis. The initial phase of LV remodeling results from fibrotic replacement of the necrotic area with scar formation, loss of myocytes and thinning of the infarcted zone^14^. Since most of the subjects in our study have CAD, the majority of LGE or scar is located at the endocardium, so that the presence of LGE is directly associated with loss of TPM mass and decreased TPMm/LVM. Beyond the early stage, the remodeling process is driven predominantly by myocyte hypertrophy, elongation and interstitial fibrosis in the non-scar zone, resulting in the increased ventricular mass, chamber enlargement and progressive decline in ventricular performance. Our results showed that the ECV, an indicator of diffuse fibrosis in non-scar area, is positively associated with TPMm/LVM indicating that the proportion of TPM mass is increased in the late phase of remodeling. In our analyses, increased ECV is also significantly associated with elevated TPM mass index but this association becomes borderlinely significant after the adjustment of LV mass.

Cardiac trabeculation occurs during the embryonic development of the heart presumably to increase the surface area to allow the myocardial mass to grow in the absence of a developed coronary circulation. The presence of trabeculae might contribute to a better mechanical efficiency of LV. Mathematical model has demonstrated that ventricular model with trabeculation is characterized by a higher compliance with respect to the smooth model^12^. Also, modeling shows that LV trabeculae contributes to the maintenance of higher cardiac output with similar atrial pressure and during faster heart rate^12^. The significant increase in TPM mass and it’s proportion to LVM in patients with SHF might be a remodeling process aiming to increase the LV mechanical efficiency, counterbalancing the deteriorated heart function.

In accord with the finding that LV trabeculation mass increases during remodeling process, another marker of trabeculaton complexity, FD is also demonstrated to increase with enlarged chamber but not with global LV systolic function in this study. FD is a highly reproducible index of trabecular complexity capable of detecting subtle subclinical trabecular phenotypes^15^. FD solely evaluates the extent to which endocardial contours fill two-dimensional space and is not influenced by the wall thickness as the other trabeculation marker such as noncompacted-to-compacted wall thickness ratio. It has been studied in patients with hypertrophic cardiomyopathy^15^ and non-compaction cardiomyopathy^5^ but never been evaluated in usual HF patients. In a large cohort enrolling subjects mainly with normal LVEF, FD is shown to be lower in Chinese American participants and is influenced by cardiac loading conditions^8^. The reference value for FD in healthy Chinese is 1.197 ± 0.070, much lower than the value in our non-HF group, presumably due to high prevalence of HTN and other comorbidities in our non-HF subjects. It is possible that the trabeculae complexity increased with elevated ventricular loads and chamber size in the early phase but remained unchanged with the deteriorated LV systolic function during LV remodeling. We also defines that FD value in patients with usual forms of SHF is around 1.27 (1.24**~**0.29) which is lower than the reference value in patients with non-compaction cardiomyopathy (1.392 ± 0.010) in literature^5^.

Our study also showed that LV trabeculation is associated with subjects’ demographics. For example, we found that the TPM mass index is positively correlated with male gender. The TPMm/LVM also has similar associations but the significance diminishes after the adjustment of other confounders. This result is similar to a large cohort with relative healthy participants^7^ and to other report using different method for trabeculae quantification^16^. For FD, our data showed that the FD is not different between genders, which is similar to the result of the sub-study of a large cohort^8^.

### Study limitations

There are several limitations of the study. First, the study population is small. Second, this study design was cross-sectional, we could not clarify the causal relationship between LV trabeculation and fibrotic repair during LV remodeling.

## Conclusions

LV TPM increases in patients with SHF probably related to myocardial cell hypertrophy and fibrotic repair during LV remodeling. The FD increases with dilatation of LV chamber but remains unchanged with the deterioration of LV function.

## Methods

### Ethics Statement

The research was approved by the institutional review board of the National Taiwan University Hospital Ethics Committee. The study was conducted in accordance with the approved guidelines. All study participants provided written informed consent.

### Patient population

The study enrolled patients with chronic HF from November 1, 2012 to July 31, 2015. Patients who met the following criteria were enrolled as the HF group: 1. they had HF symptoms of New York Heart Association (NYHA) classification functional Class II to III or a history of HF symptoms/signs by Framingham criteria^17^, and 2. the symptoms and signs of HF persisted for more than 3 months. From the HF group, diagnosis of HFpEF was defined according to the current consensus statement of the European Society of Cardiology^18^ and the ACCF/AHA task force^19^. For the diagnosis of HFpEF all of the following diagnostic criteria had to be fulfilled: 1. an echocardiographic left ventricular ejection fraction (LVEF) ≥50% and a LVEDVi ≤97 ml/m2 2. evidence of abnormal left ventricular relaxation, filling or diastolic stiffness. Pulsed-wave Doppler and Tissue Doppler Imaging were performed to obtain the ratio of early transmitral blood velocity (E) to early diastolic mitral annular velocity (e′). HFpEF was considered likely in patients with an E/e′ ratio >15 and unlikely in patients with an E/e′ ≤8. In intermediate cases with 15 > E/e′ ≥ 8, serum N-terminal prohormone of brain natriuretic peptide (NT-proBNP) levels were determined and if NT-proBNP levels exceeded 220 pg/ml, HFpEF was considered. Patients with LVEF below 50% were defined as the systolic heart failure (SHF) group. Subjects without a history of symptoms/signs were recruited as a non-HF group. Patients were excluded from the study if they had significant valvular heart diseases indicated for percutaneous or surgical intervention, chronic atrial fibrillation, chronic pulmonary disease, acute myocarditis, hypertrophic cardiomyopathy, active myocardial ischemia defined by a positive stress test or un-revascularized significant (70%) stenosis in coronary arteries by angiography, or estimated glomerular filtration rate (GFR) <30 mL/min/1.73 m^2^.

### Imaging Acquisition

MRI was performed on a 3 T MRI system (Trio, Siemens, Erlangen, Germany) with an 8-channel cardiovascular phased array torso coil. Myocardial T1 mapping was performed with an ECG-triggered Modified Look Locker Inversion recovery (MOLLI) sequence before and 10 minutes after a 0.15 mmole/kg intravenous administration of the gadolinium-based contrast agent (Omniscan, Winthrop Laboratories, GE, NJ). The MOLLI protocol used two Look-Locker cycles to acquire 7 images over 11 heart beats, the scanning parameters were TR/TE, 1.9 ms/1.0 ms; flip angle, 35°; minimum inversion time, 110 ms; inversion time increment, 80 ms; matrix size, 256 × 192; slice thickness, 6 mm; spatial resolution, 1.28 mm; GRAPPA acceleration factor, 2; number of inversions, 2; images acquired after first inversion, 5; pause 4 heart beats and images acquired after second inversion, 2. Five evenly-spaced short-axis slices were acquired sequentially from the LV base to apex. After post-contrast T1 acquisition, LGE images were acquired using an ECG-triggered phase-sensitive inversion recovery (PSIR) prepared segmented fast gradient echo pulse sequence^20^ at the same short-axis slices as those in the myocardial T1 mapping to identify the focal fibrosis or scaring.

Cine MRI was performed using a segmented balanced steady-state gradient echo pulse sequence with a retrospective ECG R-wave trigger. The scanning parameters were TR/TE, 3.0 ms/1.5 ms; flip angle, 46°; matrix size, 256 × 208 and spatial resolution, 1.21 mm. Multiple short-axis slices were prescribed from the mitral orifice to LV apex with slice thickness of 8 mm and gap of 2 mm. The true temporal resolution was 63 ms and thirty cardiac phases were reconstructed retrospectively for each slice level.

### Imaging Analysis

Myocardial ECV is an indicator of myocardial interstitial diffuse fibrosis^21^. Quantitative analysis of myocardial ECV was performed on T1 maps. The regions of interest (ROI) in the blood and the myocardium of the LV were drawn in the central area of LV cavity and the septal myocardium on T1 maps for each slice, respectively. If the septal myocardium showed regional hyperenhancement on the LGE images, the ROI of the myocardium was re-drawn in other unenhanced myocardial regions. The averaged T1 values of the segmented ROIs were then computed. After subtracting the pre-contrast values from the post-contrast values, the changes of the relaxation rate (1/T1) in the blood and in the myocardium were obtained. Myocardial ECV values were calculated using the ratio of the change in relaxation rate in the myocardium to that in the blood and multiplied by (1- hematocrit). After excluding myocardium areas with LGE, we averaged each myocardial ECV value over five short-axis slices for each subject after^22^.

For LV function and mass analysis, endocardial and epicardial contours of the LV were determined at each slice level on cine MRI and the area enclosed by each contour was computed^23^. LV volumes for each time point were then determined by the Simpson’s rule to obtain the volume-time curve of the LV. LVEDV and LVESV of the LV were assessed from the volume–time curve for the maximal and minimal values and were used to compute LVEF. LV mass was computed as the difference between LV epicardial volume at end-diastole and LVEDV, multiplied by the density of the myocardium, 1.05 g/cc. LV volumes and mass indexed to body surface area (BSA) were also measured from LVEDV (LVEDVi), LVESV (LVESVi) and LVM (LVMi) divided by BSA.

### Calculation of trabeculae papillary muscle mass and fractal dimension

**Figure 1:**
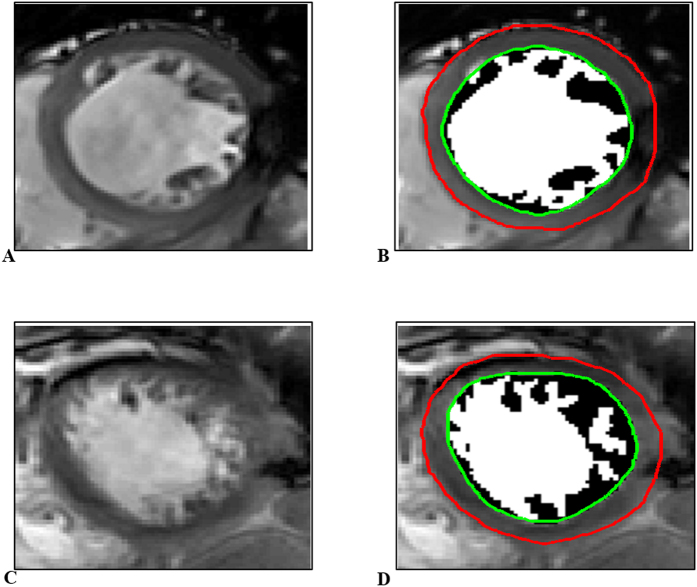
Example of trabeculae-papillary muscle quantification. (**A**) A left ventricular end-diastolic slice from a relatively trabeculated participant. (**B**) The same slice after endocardial and epicardial LV contours are delineated and trabeculae and papillary muscles (TPM) are segmented; TPM are shown in black and the residual blood pool is shown in white in (**B**). (**C**,**D**) A more heavily trabeculated participant case.

The analyses were performed by our in-house tool coding in Matlab environment (Version 7.0, MathWork Inc., Natick, MA). For TPM mass quantification, epicardial and endocardial left ventricle contours were first segmented by an automated contour detection algorithm which was presented in our previous work^24^. TPM regions located inside the endocardium were identified by a region based level set model^25^. The level set model used for segmentation of TPM only uses intensity information from endocardial regions to guide the motion of the initial contour. Once the evolution of the initial contour was converged, the TPM segmentation process was completed. Two examples of TPM quantification are presented in Fig. 1, in which the black regions inside endocardium are TPM, meanwhile the white regions inside endocardium are residual blood pool.

**Figure 2:**
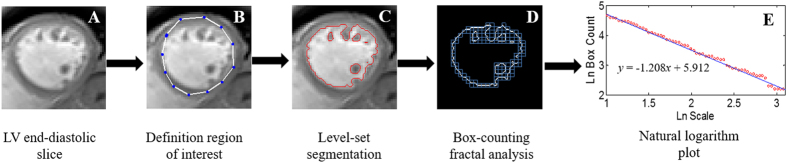
Fractal analysis procedure. A single magnetic resonance imaging (MRI) slice (**A**) from the data set. The region of interest (ROI) was defined by the user (**B**); endocardial borders were extracted using a region-based level set segmentation model (**C**). This undergoes box-counting fractal analysis (**D**): a series of grids were placed over the ROI and boxes containing non-zeros pixels were counted. This process was repeatedly applied for multiple grids with increasing spacing. A natural logarithmic plot (**E**) of box-count (y axis) against scale (x axis) was created. The slope of the line-of-the-best-fit across the points was the FD. In this example, the FD for this slice is 1.208.

In this study, fractal analysis was executed on the end-diastolic frames of each short-axis slice section. For each slice, the analysis procedure includes three steps: First, defining ROI; Second, extracting endocardial border via performing an image segmentation algorithm; Third, calculating the FD using box-counting approach. The ROI, in the first step, was selected by the user via interaction with the interface of the in-house tool. The endocardial borders were results of the segmentation step which was conducted by a region-based level set approach^26^. Especially, in this study, image segmentation and bias correction tasks for each slice were interleaved performed, which makes the segmentation yield high accuracy. The obtained endocardial border was then underwent the box-counting algorithm^5^,^27^, in which a grid with known spacing was laid over the binary endocardial border. Then the numbers of the box with nonzero pixels were counted. This procedure is repeatedly applied for multiple grids with increasing spacing. Finally, a natural logarithmic plot of box-count (y axis) against scale (x axis) is plotted and the slope of a line obtained by linear regression is derived. The slope represents a FD that is a unitless measure index of how completely the object fills space. Maximal apical FD derived from the apical half of the LV stack was chosen as a representative of endocardial complexity as previous studies^5^,^8^. An illustrated for fractal analysis procedure is presented in Fig. 2.

### Statistical analysis

Since Shapiro-Wilk test showed that some of the variables were not normally distributed, all the statistical analyses were performed by non-parametric methods. Continuous variables were expressed as medians and interquartile ranges (IQR) and categorical variables were expressed as percentages. Categorical variables were compared among different groups of patients by using Chi-square tests. Continuous variables were tested by the nonparametric Kruskal-Wallis test and the Mann-Whitney U test was used for post-hoc analysis for comparison of the medians between different groups. To investigate the determinants of LV trabeculation, a multiple linear regression model was constructed to analyze the relationship between TPM mass index, TPMm/LVM, FD and other MRI parameters since the distributions of these three parameters are similar to a normal distribution. The model was adjusted for age, gender, history of hypertension (HTN) and DM, etiology of HF (CAD vs, non-CAD), presence of LGE on MRI LGE images, LVMi, LVEDVi, ECV and LVEF. LVMi, LVEDVi, ECV were categorized into tertiles. To avoid model-dependent association, an association was considered significant only if both the univariate and multivariate model showed significant correlation. A Bonferroni test was performed for variables categorized into more than three groups to avoid multiple comparison bias. A value of P < 0.05 was considered significant. Statistical analyses were performed using the SPSS software package, version 19 (SPSS, Chicago, IL, USA).

## Additional Information

**How to cite this article**: Lin, L.-Y. *et al.* Endocardial Remodeling in Heart Failure Patients with Impaired and Preserved Left Ventricular Systolic Function-A Magnetic Resonance Image Study. *Sci. Rep.* **6**, 20868; doi: 10.1038/srep20868 (2016).

## Figures and Tables

**Table 1:** Basic demographics of the studied subjects.

Patient characteristics	SHF (n = 74)	HFpEF (n = 65)	non-HF (n = 61)
Age, year	64.0 (55.0~71.3)*	71.0 (60.0~77.5)	65.0 (60.0~71.5)
Gender (male), %	83.8*,†	46.2	45.9
BMI, kg/m^2^	24.6 (22.6~26.9)†	25.7 (22.4~28.9)	26.5 (25.0~29.6)
BSA, m^2^	1.74 (1.65~1.88)	1.67 (1.59~1.82)	1.73 (1.62~1.81)
Comorbidities			
HTN, %	82.4	83.1	85.2
DM, %	21.6	36.9	27.9
Dyslipidemia, %	45.9	38.5	52.5
CKD, %	9.5	9.2	1.6
Stroke, %	5.4	4.6	1.6
MI, %	25.7†	18.5†	4.9
CAD	50.0	40.0	44.3
PAOD, %	2.7	3.1	3.3
Valvular heart disease	4.1	3.1	1.6
Medications			
ACEI	4.1	1.5	1.6
ARB	58.1	63.1	63.9
Beta-blocker	71.6*,†	47.7	49.2

Abbreviations: SHF, systolic heart failure; HFpEF, heart failure with preserved ejection fraction; non-HF, patients without heart failure; BMI, body mass index; BSA, body surface area; HTN, hypertension; DM, diabetes mellitus; CKD, chronic kidney disease; MI, myocardial infarction; CAD, coronary artery disease; PAOD, peripheral arterial occlusive disease; ACEI, angiotensin converting enzyme inhibitor; ARB, angiotensin receptor blocker.

^*^p < 0.05 compared with HFpEF.

^†^p < 0.05 compared with the non-HF group.

**Table 2:** Magnetic resonance image parameters in patients with and without heart failure.

	SHF (n = 74)	HFpEF (n = 65)	Non-HF (n = 61)
LVEDVi, ml/m2	107.7 (83.8~132.2)*,†	50.9 (41.5~65.2)	48.6 (39.7~57.4)
LVESVi, ml/m2	70.4 (47.5~94.1)*,†	11.2 (7.2~21.3)	10.2 (7.1~13.7)
LVEF, %	36.1 (28.6~43.1)*,†	77.3 (65.4~84.2)	78.2 (73.6~83.7)
LVMi, gm/m2	94.6 (79.2~112.4)*,†	61.0 (51.8~81.1)	58.8 (52.4~74.5)
LGE, %	67.1%*,†	41.5%†	14.8%
ECV, %	32.5 (28.6~43.1)*,†	30.7 (28.6~32.5)†	27.8 (25.6~29.3)
TPMi, g	24.1 (18.6~31.3)*,†	13.8 (11.9~17.7)	13.3 (11.2~17.9)
TPMm/LVM, %	25.7 (22.0~29.8)*,†	23.7 (20.1~25.2)	23.1 (20.4~25.0)
FD	1.27 (1.24~1.29)	1.26 (1.23~1.30)	1.25 (1.21~1.29)

Abbreviations: SHF, systolic heart failure; HFpEF, heart failure with preserved ejection fraction; non-HF, patients without heart failure; LVEDVi, left ventricular end-diastolic volume index; LVESVi, left ventricular end-systolic volume index; LVEF, left ventricular ejection fraction; LVMi, left ventricular mass index; LGE, late gadolinium enhancement; ECV, extracellular volume fraction; TPMi, trabeculae and papillary muscle mass index; TPMm/LVM, TPM mass and left ventricular mass ratio; FD, fractal dimension.

^*^p < 0.05 compared with HFpEF.

^†^p < 0.05 compared with the non-HF group.

**Table 3:** Multiple linear regression model to identify the determinant of trabeculae papillary mass-left ventricular mass ratio (TPM-LM ratio) and fractal dimension (FD).

	TPMi	TPMm/LVM	FD
Age, years			
60~75 vs. 60	−2.198 (−5.103~−0.707)	−0.025 (−0.047~−0.002)	0.007 (−0.010~0.024)
≧75 vs. 60	−2.885 (−6.475~−0.705)	−0.026 (−0.053~0.002)	0.001 (−0.020~0.022)
Gender (male)	3.313 (0.326~6.300)*	0.011 (−0.012~0.034)	−0.016 (−0.033~−0.001)
HTN	1.423 (−1.873~4.744)	0.017 (−0.009~0.043)	0.004 (−0.015~0.023)
DM	−1.423 (−4.085~1.239)	−0.010 (−0.031~0.010)	−0.004 (−0.019~0.011)
Etiology of HF (CAD vs. non-CAD)	−0.024 (−2.616~2.568)	0.005 (−0.015~0.025)	−0.012 (−0.027~0.003)
LGE	−5.308 (−8.482~−2.134)*	−0.026 (−0.051~−0.002)*	−0.002 (−0.020~0.016)
LVMi, gm/m2			
60.2~84.5 vs. ≦60.2	2.538 (−0.796~5.872)	−0.021 (−0.047~0.005)	0.005 (−0.014~0.025)
>84.5 vs. ≦60.2	9.924 (5.984~13.864)*	−0.024 (−0.054~0.007)	0.004 (−0.019~0.026)
LVEDVi, ml/m2			
49.9~77.2 vs. ≦49.9	0.174 (−2.859~3.208)	0.007 (−0.017~0.030)	0.028 (0.010~0.045)*
>77.2 vs. 49.9	5.060 (−0.101~10.222)	0.021 (−0.019~0.061)	0.056 (0.026~0.086)*
LVEF, %			
35~50 vs. ≧50.0	0.418 (−4.093~4.929)	0.014 (−0.021~0.049)	−0.018 (−0.044~0.008)
<35 vs. ≧50.0	4.465 (−0.727~9.658)	0.041 (0.001~0.082)	−0.041 (−0.071~−0.011)
ECV, %			
30~32 vs. ≦29	2.763 (−0.335~5.861)	0.007 (−0.017~0.031)	0.018 (0.001~0.036)
>33 vs. ≦29	3.892 (0.211~7.574)	0.035 (0.006~0.063)*	0.011 (−0.010~0.032)

Abbreviations: TPMi, trabeculae and papillary muscle mass index. TPMm/LVM, trabeculae and papillary muscle mass and left ventricular mass ratio; FD, fractal dimension; HTN, hypertension; DM, diabetes mellitus; LGE, late gadolinium enhancement; LVMi, left ventricular mass index; ECV, extracellular volume; HFpEF, heart failure with preserved ejection fraction; non-HF, patients without heart failure; SHF, systolic heart failure.
